# Hospital for Sick Children

**Published:** 1904-06-11

**Authors:** John Murray


### HOSPITAL FOR SICK CHILDREN.

*To the Editor of* THE HOSPITAL.

SIR,—In the obituary notice of Mr. Adrian Hope, which appeared in THE HOSPITAL for May 14th, I read the following statement:—"In 1888 the total revenue of the Hospital [for Sick Children] was a little over £20,000 a year, and in 1902 the total receipts exceeded £41,000," etc. As this statement is somewhat misleading, and calculated to produce an impression that we are much richer than we are, may I be allowed to tell your readers that in 1888 our "revenue . . . a year"—which I take to mean more or less reliable ordinary income—was £13,239. The proceeds of the Children's Jubilee Tribute and legacies brought in an additional sum of £7,821, but this can hardly be reckoned as revenue. In 1902 the Imperial Coronation Bazaar produced a sum of £17,852 18s. 1d.; not £19,000, as stated in your article. I am, dear Sir,

Yours faithfully,
JOHN MURRAY.

